# Idiopathic hypereosinophilic syndromes and rare dysimmune conditions associated with hyper-eosinophilia in practice: An innovative multidisciplinary approach^☆^

**DOI:** 10.1016/j.waojou.2024.100928

**Published:** 2024-07-25

**Authors:** Marco Caminati, Lucia Federica Carpagnano, Chiara Alberti, Francesco Amaddeo, Riccardo Bixio, Federico Caldart, Lucia De Franceschi, Micol Del Giglio, Giuliana Festi, Simonetta Friso, Luca Frulloni, Paolo Gisondi, Mauro Krampera, Giuseppe Lippi, Claudio Micheletto, Giorgio Piacentini, Patrick Pinter, Maurizio Rossini, Michele Schiappoli, Cristina Tecchio, Laura Tenero, Elisa Tinazzi, Gianenrico Senna, Matilde Carlucci, Multidisciplinary Group on rare dysimmune conditions with hyper-Eosinophilia (GEos)

**Affiliations:** a Department of Medicine, University of Verona, Verona, Italy; b Allergy Unit and Asthma Center, Verona Integrated University Hospital, Verona, Italy; c University of Verona, Verona Italy; d Pharmacy Unit, Verona Integrated University Hospital, Verona, Italy; e Unit of Psychosomatics and Medical Psychology, Verona Integrated University Hospital, and Department of Neurosciences, Biomedicine and Movement, University of Verona, Italy; f Rheumatology Unit, Verona Integrated University Hospital, Verona, Italy; g Gastroenterology and Digestive Endoscopy Unit, The Pancreas Institute, Verona Integrated University Hospital, Verona, Italy; h Unit of Internal Medicine B, Verona Integrated University Hospital, Verona Italy; i Section of Dermatology and Venereology, Department of Medicine, University of Verona, Verona, Italy; j Pulmonology Unit, Verona Integrated University Hospital, Verona Italy; k Department of Engineering for Innovation Medicine, Section of Innovation Biomedicine, Hematology Area, University of Verona, Verona, Italy; l Section of Clinical Biochemistry and School of Medicine, University of Verona, Verona, Italy; m Department of Surgical Sciences, Dentistry, Gynecology and Pediatrics, Pediatric Clinic, University of Verona, Verona, Italy; n Unit of Otolaryngology, Head and Neck Department, University of Verona, Verona, Italy; o Health Directorate, Verona Integrated University Hospital, Verona, Italy

**Keywords:** HES, Hypereosinophilic syndrome, Mepolizumab, Benralizumab, Multidisciplinary, Precision medicine

## Abstract

Hypereosinophilic syndromes (HES) represent a group of rare dis-immune conditions characterized by blood hyper-eosinophilia and eosinophilic related burden. Especially the idiopathic subtype (I-HES) is particularly difficult to diagnose because of its heterogeneous clinical presentation, the lack of specific findings on physical exam, lab tools, and imaging informative enough to unequivocally confirm the diagnosis and the overlap with other entities, including eosinophilic organ-diseases or systemic dis-immune conditions other than I-HES (from atopy to eosinophilic granulomatosis with polyangiitis [EGPA], the last often extremely difficult to distinguish from HES). Taken together, all the features mentioned above account for an extremely difficult early recognition HES and on-time referral to a specialized centre. The referral itself is challenging due to a not univocal specialist identification, because of the variability of physicians managing HES in different settings (including allergist/clinical immunologist, haematologist, internal medicine doctors, pulmonologist, rheumatologist). Furthermore, the approach in terms of personalized treatment identification and follow-up plan (timing, organ assessment), is poorly standardized. Further translational and clinical research is needed to address the mentioned unmet needs, but on practical grounds increasing the overall clinicians’ awareness on HES and implementing healthcare pathways for HES patients represent a roadmap that every clinician might try to realize in his specific setting.

The present review aims at providing an overview about the current challenges and unmet needs in the practical approach to HES and rare hypereosinophilic allergo-immunological diseases, including a proposal for an innovative multidisciplinary organizational model.

## Introduction

Despite their limited epidemiological “size” rare diseases are characterized by relevant burden in terms of patients’ quality of life and morbidity, which is further amplified by their difficult early recognition, the diagnostic challenges and by the poor availability of specific or targeted treatments.^1^

**Fig. 1:**
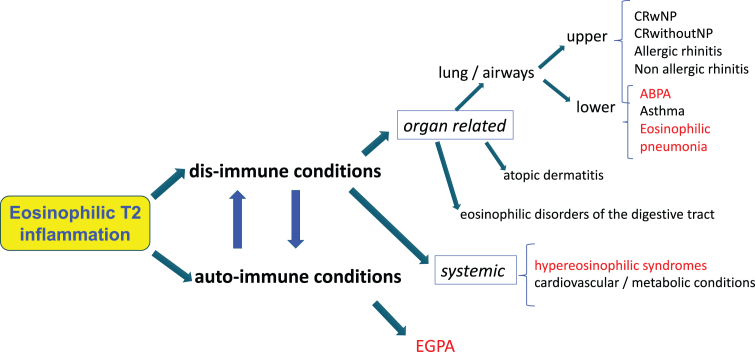
Overview of the most relevant immunological conditions sharing a T2-eosinophilic inflammation in their pathobiological background still with different clinical manifestations and burden. Red colour indicates systematic association with blood hypereosinophilia. ABPA: allergic bronchopulmonary aspergillosis. CRSwNP: chronic rhinosinusitis with nasal polyps. EGPA: eosinophilic granulomatosis with polyangiitis.

In the field of Allergy and Clinical Immunology the recent advances in the knowledge of pathobiological mechanisms and the development of new-targeted drugs are contributing to polarize the interest and attention of clinicians about rare immunological conditions characterized by hyper-eosinophilia and sharing a common T2 inflammatory background. That is the case of hypereosinophilic syndrome (HES), eosinophilic esophagitis (EoE) and other eosinophilic disorders of the digestive tract (EGIDs), eosinophilic lung diseases (allergic bronchopulmonary aspergillosis - ABPA, acute/chronic eosinophilic pneumonia – EP), and eosinophilic granulomatosis with polyangiitis (EGPA)^2^ (Fig. 1).

**Fig. 2:**
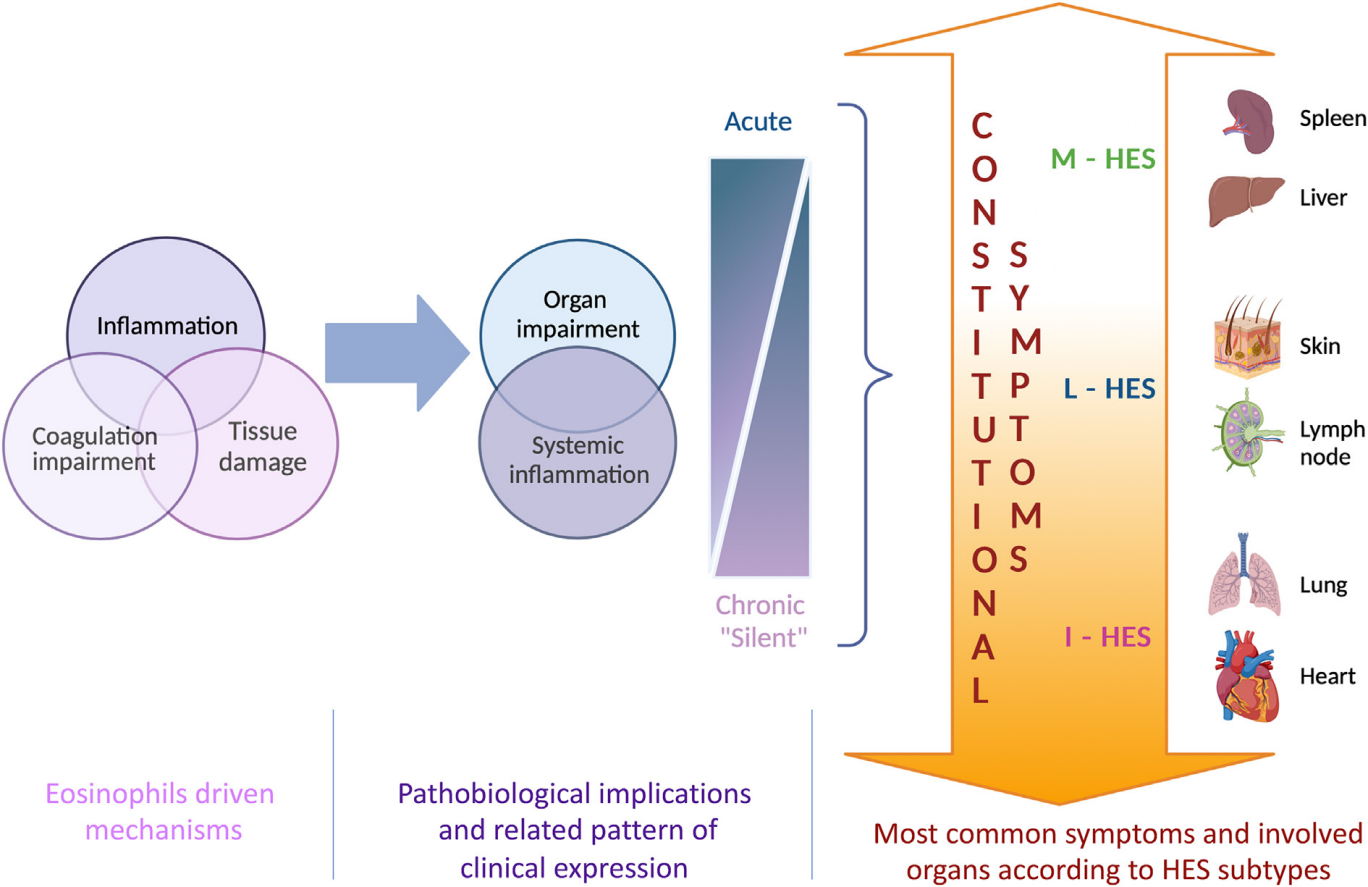
Schematic description of eosinophils driven mechanisms in HES, their pathobiological implications and related patterns of clinical expression and of the most common symptoms and involved organs according to HES subtypes. I-HES: idiopathic hyperesoniphilic syndrome; L-HES: lymphocytic hyperesoniphilic syndrome; M-HES: myeloid hyperesoniphilic syndrome

Under a pathobiological perspective, the central role of eosinophils in all the above-mentioned conditions has supported the “eosinophilic march” concept, describing the potential trajectory of T2 disorders, from allergic rhinitis to hypereosinophilic syndrome according to the amount and burden of blood and/or tissue eosinophils.^3^ That perspective, although valuing on a clinical ground the pleiotropic clinical expression of eosinophilic inflammation, only partially reflects the complexity of the different immunological scenarios sharing an increased eosinophilia. In fact, the well-known T2 inflammation may overlap with a typical autoimmune environment, like in the case of EGPA,^4^ or with an epithelial-driven cascade expressing a functional and anatomical barrier dysfunction, as in the case of EoE.^5^ A combined inflammatory and cytotoxic eosinophilic-mediated impairment along with potential impact of eosinophils on coagulation homeostasis probably characterize HES dis-immunity^6^^,^^7^ (Fig. 2).

However, the strong evidence in favour of highly selective monoclonal antibodies targeting T2 inflammation and previously licensed for severe asthma has paved the way to the investigation and approval of some of those options for EGPA, EoE, and I-HES.^8^

The availability of targeted treatments certainly contributes to improve the overall management of rare hypereosinophilic allergo-immunological diseases, which are no more orphan, but at the same time, further sustains the need for appropriate and updated healthcare pathways in order to shorten the patient's journey, properly manage the disease complexity and overall improving the standard of care for affected patients.

The present review aims at providing an overview about the current challenges and unmet needs in the practical approach to HES and rare hypereosinophilic allergo-immunological diseases, including a proposal for an innovative multidisciplinary organizational model.

## Practical tools and challenges in diagnosis

### Clinical

Suspecting HES, and even more idiopathic HES (I-HES), when evaluating a patient is more than challenging. In fact, eosinophil-mediated pathobiological burden might potentially involve every organ, which accounts for the great variability of its clinical presentation. The organ damage occurring in the context of HES is usually the result of a long history of persistent eosinophilic tissue infiltration, which is typically silent at the first disease stages, although an acute presentation is also possible. The consequent fibrotic evolution is responsible for a progressive dysfunctional impairment, which sustains the clinical manifestations affecting heart, lung, digestive tract, skin, peripheral or central nervous system, vascular apparatus (including both vasculitis and thrombosis), and, to a lesser extent, pancreas, kidney, liver, spleen.^9^^,^^10^

In addition to the wide and poorly specific spectrum of organ-related manifestations, a further challenge in HES patients’ recognition is the recurrence of constitutional symptoms, which by definition are not suggestive of a specific condition but may represent the only clinical appearance of the disease especially in I-HES patients. Fatigue, recurrent fever, malaise, myalgia, which by definition are poorly suggestive of a specific condition, occur as a “silent” but persistent impairment daily affecting HES patients.^11^

Some attempts have been provided to identify a clinical fingerprint for each HES subtype (Fig. 2). Requena et al have analysed 171 articles containing data on 347 separate HES cases, published between 2000 and 2020. Studies reporting secondary, associated/reactive, overlap/single-organ, or familial HES were excluded.^12^ According to the results, up to 79.0% of patients with lymphocytic HES (L-HES) present with skin manifestations. Other common abnormalities in L-HES patients do include bone-marrow (41.9% of patients), and lymph nodes (33.9% of patients). Regarding skin manifestations, they include: angioedema, with or without concomitant wheals commonly a bilateral peri-orbital swelling, sometimes with delicate hyper-pigmentations; urticaria-like lesions; cutaneous erythematous swelling resembling bacterial cellulitis and Well's syndrome; pruriginous papules and papulo-necrotic lesions presenting as erythematous pruritic papules and nodules; eczematous lesions, which may occur anywhere and present as persistent, erythematous, thin and scaling plaques, resembling atopic eczema with severe pruritus; mucosal ulcers; and more rarely, splinter haemorrhages and/or nail-fold infarcts, which may predict thromboembolic complications and eosinophilic endo-myocardial involvement.

In the case of myeloid HES (M-HES), the most common presentation was splenomegaly (64.5% of patients), bone marrow abnormalities (36.4%), and heart and liver impairment (both 29.8%), which is not surprising when considering that M-HES is a clonal subtype, defined as eosinophilic myeloid neoplasm, thus more similar in its presentation to haematological malignancies.

On the opposite Requena et al^12^ confirmed a wide spectrum of potential clinical manifestations in the case of idiopathic-HES (I-HES), being the most frequent related to heart (34.9% of patients), bone marrow (34.9%), and lung (33.6%).

Endo-myocardial damage is a well-known complication in I-HES patients and is commonly a leading cause of death. Restrictive cardiomyopathy, heart failure, intra-cardiac thrombosis, valve involvement due to sub-valvular thrombi, and subsequent fibrotic evolution, arrhythmias, pericardial effusion and pericarditis have been described in HES patients.^13^ The evolution of cardiac damage is usually characterized by 3 stages.: The acute phase is characterized by the eosinophilic cytokines-mediated damage to the endocardium. This phase is often asymptomatic, so troponin T (characteristically increased since the onset of the disease even with echocardiogram still in the norm), NT-pro-BNP, C reactive protein (signs of miocardiocytic damage), and echocardiogram are critical. The thrombotic stage usually develops after 10 months, where a layered thrombus forms due to activation of tissue factor by the eosinophils. The fibrotic stage occurs after 24 months, with myocardial fibrosis causing wall stiffness.^13^^,^^14^ In addition, eosinophils have significant pro-aggregation and procoagulant properties, which may cause venous or arterial thrombosis, vasculopathy, eosinophilic vasculitis, arterial and venous aneurysms, acral ulcers, Raynaud's phenomenon, or coronary artery spasm in HES.^7^^,^^13^^,^^15^

Eosinophilic infiltration can occur at various levels of the airways and appear as rhinosinusitis and nasal polyposis, asthma, bronchial hyper-responsiveness, and sub-epithelial fibrosis.^16^ When eosinophilia extends to alveolar spaces and lung interstitium, eosinophilic pneumonia occurs, and if the infiltration involves a large area of parenchyma it will appear exchange impairment with hypoxemia.^17^ This impairment could occur also when eosinophils accumulate within the pleural space, leading to eosinophilic pleural effusions and subsequent compression of the lung.^18^ Duncan et al^19^ also demonstrated that eosinophilic inflammation appears to be very important in the context of mucus plugging in airway disease, with patients who had the highest grade of mucus plugging also having the highest levels of airway eosinophilia.

Although not listed among the most recurring presentations, gastrointestinal manifestations deserve to be considered as they represent a common reason leading the patient to visit a doctor or the Emergency Room.^20^^,^^21^ Due to the heterogeneous nature of HES, especially I-HES, a broad constellation of clinical signs and symptoms can occur. Upper digestive tract symptoms like dysphagia, heartburn, chest pain, or food impaction are more frequently suggestive of single organ eosinophilic disease (eosinophilic esophagitis or gastritis), although eosinophilic colitis or enteritis with related symptoms are also possible, lower digestive tract manifestations more commonly associate with HES. They include abdominal pain, nausea, dyspepsia, vomiting, diarrhoea, as acute presentation and malabsorption, hypo-albuminemia, anaemia, and weight loss as signs of chronic organ involvement. In addition, patients may present with obstruction or intestinal intussusception if eosinophilic infiltrate affects the muscle layer, or with peritonitis, ascites, and perforation in the case of serosa layer involvement.^22^

Other clinical manifestations may be represented by: ocular involvement which may present as blurry vision caused by local micro thrombi or micro embolic phenomena, or also choroid abnormalities;^23^ musculoskeletal involvement, which may present as arthralgia, arthritis, effusions of the large joints, myalgia, even if myositis and poly-myositis are rare;^9^^,^^24^ neurological involvement, which may present as primary generalized central nervous system dysfunction, thromboembolic phenomena affecting the central nervous system, and peripheral neuropathies, caused by eosinophil-derived neurotoxin that direct nerve damage or by eosinophil-mediated damage to endothelial cells that leads to edema and subsequently pressure on nerves with axonal damage;^9^^,^^25^ and kidney involvement, which is dominated by nephrotic syndrome as main clinical manifestation, with or without renal insufficiency. Renal damage can occur in many different pathological types which include IgA nephropathy, membranous nephropathy, podocyte disease, focal segmental glomerular sclerosis, chronic interstitial nephritis, membrane hyperplasia lesion, mesangial proliferative lesion, crescent glomerulonephritis, thrombotic microangiopathy, and immunotactoid glomerulopathy.^26^

Generally speaking, depicting a typical clinical profile related to HES might be misleading. However, as respiratory symptoms and skin lesions are common reasons leading the patients to visit an allergist/clinical immunologist or a respiratory medicine doctor, or a dermatologist, all of them should be ready to recognize or at least suspect an underlying HES in the background of the patient's manifestations. Of note, especially in the case of skin manifestations or splenomegaly/lymph nodes involvement, L-HES and M-HES respectively are likely, and as they are the HES subtypes closest to haematological malignancies they must be excluded.

## Blood hyper-eosinophilia and eosinophils related burden demonstration

In up to 10% of patients presenting blood hyper-eosinophilia, that condition is completely asymptomatic.^10^ How to manage hyper-eosinophilia of unknown significance, especially in terms of treatment and follow-up, currently represents one of the major controversial aspect, as no information are available so far about its potential trajectory.^10^^,^^27^^,^^28^

However, at the moment, this is the reason why HES diagnosis cannot be confirmed on a blood sample only. On the opposite, due to its heterogeneous and poorly specific clinical profile, HES suspicion cannot rely on symptoms only and must be ruled out in the absence of a suggestive blood eosinophil count.^10^^,^^29^

In fact, even in the case of clear organ related-symptoms, which can be more easily recognized, defining the diagnosis might be challenging and requires the distinction between an organ eosinophilic disease (ie, EGID, EP, eosinophilic bronchitis, asthma) and HES with organ manifestations, the so-called overlapping HES according to the classification by Klion.^10^ The detection of blood hyper-eosinophilia (namely blood eosinophils >1500 cells/microliter) on two consecutive occasions persistent for a minimum of 1 month^10^^,^^29^ makes the difference, representing a major diagnostic criterion for HES. Of course, the evaluation of blood eosinophils should be performed in the absence of a potentially biasing pharmacological treatment (ie, oral steroids), and should be repeated in the case of clinical suspect and increased eosinophil count although not matching the diagnostic threshold.

However, an increase in blood eosinophils above 1500 cells may occur in some eosinophilic organ diseases, including active ABPA, acute EP or the same uncontrolled eosinophilic asthma, and in the case of a systemic eosinophilic condition other than HES, namely EGPA, which still shares with HES some pathobiological and clinical features.^16^^,^^30^^,^^31^ Especially in the last case, the differential diagnosis might be extremely challenging, and however of utmost importance for the implications in terms of treatment options and follow-up that HES vs EGPA diagnosis does have.

In addition to eosinophilic organ-diseases, a number of conditions having nothing to do with HES may account for blood hyper-eosinophilia: atopic disorders, drug-related or drug hypersensitivity, infections and infestations, autoimmune disorders and immuno-deficiencies, neoplasia.^11^

In the light of that described above, once excluded known reasons for hyper-eosinophilia, the demonstration of an association between blood eosinophils increase and clinical manifestations/organ impairment should be provided.^10^^,^^29^

Under histopathologic perspective, the definition of tissue infiltration requires to match >1 of the following criteria: a) the percentage of eosinophils in bone marrow sections exceeds 20% of all nucleated cells, b) extracellular deposition of eosinophil granule proteins (as EPX or eMBP1) finding at immuno-staining, and c) the opinion of a pathologist that tissue infiltration by eosinophil is massive compared to normal physiologic ranges.^11^ However, biopsy is not always feasible and in addition its sensitivity might not be optimal due to the patchy distribution of damage.^10^ Combining clinical manifestations and blood count with imaging or other instrumental data might be helpful in corroborating the diagnosis.

Practically speaking, in the case of heart involvement is suspected, a simple electrocardiogram may reveal abnormalities; of course, it does not allow an early detection of heart damage, reflecting the expression of long-term organ impairment. The echocardiogram might be more sensitive, allowing evaluating the kinetics of the ventricles, wall thickness, mobility and valve function, areas of fibrosis, ventricular hypertrophy and presence of thrombi. However, in the initial phases even the echocardiogram can be normal, so the cardiovascular magnetic resonance (CMR) can be more sensitive in identifying the damage. CRM allows to recognize thrombi in the ventricular site and CMR with gadolinium can distinguish between inflammatory and fibrotic tissue and describe the degree of fibrosis; the pattern of late gadolinium enhancement is sub-endocardial with patchy or diffuse distribution, without association to a coronary artery distribution.^13^

Different imaging techniques such as standard chest x-ray but even more High-Resolution CT scanning, as well as bronchoscopy with broncho-alveolar lavage, support the investigation of lung manifestations. Pulmonary abnormalities usually found in HES include nodules, ground-glass opacities and consolidation in a patchy distribution, interlobular septal thickening, and pleural effusion, and increased number of eosinophils in the bronchoalveolar lavage fluid or eosinophilic infiltrate on the lung biopsy documents an eosinophilic pathogenesis.^16^^,^^32^

In order to explore digestive tract, upper and lower endoscopy might be performed, revealing erythematous, friable, ulcerated mucosa, vertical linear furrows and strictures in the oesophageal tract, and pseudo-polyps in the gastrointestinal tract. In this case biopsy is quite easy to perform; histological confirmation is given by the presence of marked extracellular deposition of eosinophil granule proteins or peak eosinophil counts higher than cut offs (≥15 eos/HPF in the oesophagus, ≥30 eos/HPF in the stomach or small bowel, ≥60 eos/Hpf in large bowel). CT and MRI may also support the diagnosis, even if imaging findings are nonspecific.^22^

Although less common neurological involvement may present as primary generalized central nervous system dysfunction, thromboembolic phenomena affecting the central nervous system, and peripheral neuropathies, caused by eosinophil-derived neurotoxin that direct nerve damage or by eosinophil-mediated damage to endothelial cells that leads to edema and subsequently pressure on nerves with axonal damage.^9^^,^^25^

## Molecular tools and additional lab findings

I-HES is not associated with known and detectable specific abnormalities at a molecular level, but performing some essential molecular assessment is the only way to exclude M-HES and L-HES, which represent the “closest” HES subtypes to a haematological malignancy. In fact, M-HES is a clonal subtype defined as eosinophilic myeloid neoplasm; L-HES is characterized by a clonal population which can be indistinguishable from that seen in T-cell malignancies, (especially angioimmunoblastic T-cell lymphoma and cutaneous T-cell lymphoma).^10^^,^^28^ In addition, progression of L-HES to a lymphoid malignancy occurs in approximately 10% of patients.^33^ Thus, if HES diagnostic criteria are matched, molecular assessment is mandatory to confirm I-HES diagnosis.

Myeloid (and lymphoid) neoplasms with eosinophilia are associated with rearrangement of the tyrosine kinase (TK) genes, namely PDGFRB, PDGFRA, FGFR1, JAK2, FLT3, and ABL1 (excluding BCR-ABL1). The presence of FIP1L1PDGFRA fusion gene is the most known abnormality, but more than 70 fusion genes involving one of these six fusion driver TK genes have been reported to date.^34^ Fused/mutated genes result in the expression of an aberrant TK or receptor TK that involve both myeloid and lymphoid lineages, and therefore also eosinophils, resulting in hyper-eosinophilia. Those abnormalities can be detected by evaluating blood samples and bone marrow. Cytogenetic analysis can identify translocations; FISH technique is able to locate rearrangements and fusions, which are cytogenetically occult; RT-PCR detects false-negative FISH results; NGS mutation panel for eosinophilic myeloid neoplasms allows catching somatic mutations.^10^^,^^34^

In L-HES one or more T cell subsets with an aberrant immuno-phenotype, with or without evidence of a clonal T cell receptor (TCR) gene rearrangement can be found. Lymphocyte phenotyping by flow cytometry can be performed on both peripheral blood and bone marrow.

Aberrant/clonal T cells commonly present the absence of CD3 (eg, CD3^−^CD4^+^, most common), a normal component of the T-cell receptor complex, or double negative, immature T-cells (eg, CD3^+^CD4^−^CD8^−^); other variants have been described including CD3^+^ CD4^+^ CD7^−^, CD3^+^ CD4^−^ CD8^−^ TCRαβ+, loss of CD7 and/or CD27^+^.^33^^,^^34^ Clonal rearrangement of T-cell receptor genes was demonstrated in half of the patient with L-HES, even if clonal T-cell receptor gene rearrangements are in general highly prevalent in patients with various HES subtypes. The presence of this variant is estimated to be 17%–27% in patients with unexplained eosinophilia or HES.^35^^,^^36^

In addition, some laboratory findings such as steroid refractory persistent/worsening eosinophilia, leucocytosis, circulating blasts, dysplastic cells, thrombocytopenia or thrombocytosis, anaemia, increase in serum vitamin B12 (>1000 pg/mL, due to the increased production of haptocorrins, which bind to vitamin B12 in serum and various tissues) or in serum triptase (>12 ng/mL) may help in differential diagnosis with malignant subtypes.^37^

## Management in practice: current options and challenges

I-HES treatment approach mostly relies on drugs coming from other indications and not specifically developed for that condition, and characterized by a non-optimal safety profile. Furthermore, the multiple eosinophil-related pathobiological mechanisms most likely underlying I-HES mentioned above, including inflammation, direct tissue damage and coagulation impairment, should be all addressed and taken into consideration when defining the long-term treatment and follow-up plan.^38^

**Fig. 3:**
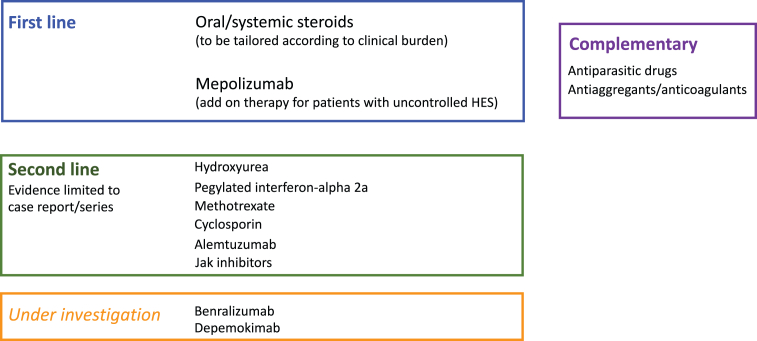
Overiew of treatment options for I-HES, according to the current regulatory setting and the reports in the literature related to the idiopathic subtype of hypereosinophilic syndrome

Fig. 3 summarizes the panorama of treatment options for I-HES, according to the current regulatory setting and the reports in the literature related to the idiopathic subtype of HES.

Steroids still represent the first line option both for the management of acute onset, if occurring, and for the remission maintenance.^10^^,^^28^

In case of severe life-threatening eosinophilic respiratory, vascular, cardiac or neurological involvement, even in the case the differential diagnosis within HES subtypes has not been confirmed, the initial management is based on corticosteroid therapy with 1 mg/kg/day prednisone, preceded by intravenous pulses of methylprednisolone 5–15 mg/kg/day up to a maximum of 1000 mg for 3 days. Systemic steroid therapy may also be considered in case of severe thrombosis, given its ability to induce rapid normalization of eosinophilic counts.^7^^,^^13^

In long-term management, the minimal effective dose sustaining the remission maintenance, defined as absence of HES-related clinical symptoms and a peripheral blood eosinophil count increase, should be identified. On-demand administration with short courses is also an option in case of transient recurrent manifestations.^10^^,^^28^

According to the recently published French guidelines on HES,^28^ antiparasitic and anticoagulation treatment should be combined with steroids in the management of acute onset of HES.

The place of empirical antiparasitic treatment in currently under debate; however its rationale relies on 2 main points: accuracy of blood and stool parasitological investigations is generally speaking limited so the exclusion of a parasitic infection as an underlying cause of hyper-eosinophilia, cannot be definitive; and high dose systemic corticosteroids might amplify the burden of an unrecognized parasitic infection. The excellent tolerability profile and the low cost of antiparasitic drugs further sustain its use.^28^ Albendazole, at a dose of 10–15 mg/kg/day up to a maximum of 800 mg/day, taken twice daily with meals for 10–15 days, is a good treatment option. In general, before starting steroid therapy, it is recommended pre-emptive treatment with ivermectin, at a dose of 200 μg/kg on an empty stomach, for prophylaxis of severe Strongyloidiasis infection.^28^

Regarding anticoagulation treatment, it addresses the potential impact of eosinophils on coagulation homeostasis. In fact, increased eosinophil count has been described as an independent risk factor for recurrence of venous and arterial thrombosis in a multivariate analysis performed in a retrospective study of 54 patients with venous thromboembolism secondary to hyper-eosinophilia.^7^ Which would suggest the anticoagulation prophylaxis in hyper-eosinophilia of unknown significance, besides HES. No data in favour of vitamin K antagonists or direct anticoagulants are available so far. Antiplatelet agents should be considered especially in these case of concomitant cardiovascular risk factors.^7^^,^^28^

Second-line treatments for I-HES, sustained by limited evidence, include: Hydroxyurea, 1–2 g/daily, oral, with anti-eosinophilic activity due to its non-specific myelotoxic activity; and interferon-alpha 2a, 1–3 mU daily or 3 times per week, subcutaneous (available only in its pegylated form), which acts on both eosinophilia and T2-polarized T cells. Alternatively, immunosuppressive agents including methotrexate (7.5–20 mg weekly, oral or subcutaneous) and cyclosporine (150 mg daily, oral) can be considered, although a weak evidence is available so far.^10^^,^^38^

Very recently, mepolizumab 300 mg/4 weeks, subcutaneously, has been licensed in the United States and Europe as an-add on therapy for patients with uncontrolled HES without an identifiable non-hematologic secondary cause.^39^^,^^40^ Mepolizumab selectively interferes with IL-5 cascade, the most relevant pathway for eosinophils' generation, development and survival, so it has the chance to specifically address the major driver of I-HES pathobiological background with a consistent-steroid sparing agent. Its placement, in terms of timing in respect of (acute) disease onset and association with other drugs has to be clarified in clinical practice. However, it is the first drug for I-HES that has been tested in a randomized clinical trial specifically including I-HES patients,^39^ demonstrating a significant impact on disease flares, fatigue, oral steroid dependence and blood eosinophil count. In addition, its safety and efficacy profile has been proved by years of experience in severe eosinophilic asthma patients, although at the dose of 100 mg/4 weeks.^41^ Some emerging real-life data suggest a room for personalizing mepolizumab treatment by tailoring the dose (300 mg/4 weeks vs 100 mg/4 weeks) according to the disease stage (remission induction vs remission maintenance) and patient's profile.^12^^,^^21^^,^^42^^,^^43^ After all, it's not out of place when considering that the identification of 300 mg as optimal dose for HES does not result from a true dose-finding investigation but is justified by the principle that the higher the baseline blood eosinophil count, the higher the mepolizumab dose required to achieve the same absolute in-range target counts.^44^

Other targeted therapies for I-HES are currently under investigation. A phase 3 trial,^45^ following a phase 2 clinical trial that have shown promising results in HES^46^ is ongoing about the use of benralizumab, a monoclonal antibody targeting the alpha subunit of IL-5 receptor (IL-5Rα) and depleting eosinophils and their precursors by Ab-dependent cell-mediated cytotoxicity. Depemokimab, a long-acting IL-5R antagonist mAb, is under investigation in a randomized double-blind placebo-controlled study currently ongoing, to investigate its efficacy and safety in adults with uncontrolled HES.^47^ Alemtuzumab was explored some years ago in case reports and small series,^48^, ^49^, ^50^ and more recently described in a large real-life US cohort.^12^ Dupilumab have also demonstrated benefit in the treatment of some patients with HES.^51^

Independently of the treatment, a crucial point in I-HES patients is long-term follow up. In fact, the disease trajectory is not known at the moment so the potential evolution towards clonal subtypes cannot be fully excluded even in the case of confirmed I-HES. Furthermore, blood eosinophils count does not accurately parallel tissue eosinophilia, so that even in the case of maintained in-range blood eosinophilia organ assessment should be periodically be performed, including the most critical districts such as heart and lung.

## Addressing I-HES unmet needs starts from patient recognition

Rare diseases share a number of practical challenges related to the limited expertise on their recognition and management each physician has the chance to acquire, due, by definition, to their low prevalence.^1^ As described above, I-HES, but more in general hyper-eosinophilic syndromes, is particularly difficult to diagnose because of its heterogeneous clinical presentation, the lack of specific findings on physical exam, lab tools and imaging informative enough to unequivocally confirm the diagnosis and the overlap with other entities, including eosinophilic organ-diseases (EGIDs, CRSwNP, asthma) or systemic dis-immune conditions other than I-HES (from atopy to EGPA, the last often extremely difficult to distinguish from HES). For the same reasons, the currently available HES epidemiological data are very variable, ranging between 0.15 and 6.3 cases per 100,000 people across different countries and settings.^12^^,^^29^^,^^52^ In addition, in the absence of acute onset or in the case acute onset is not recognized as HES, the clinical appearance of the disease may occur after long time from the beginning of blood hyper-eosinophilia when an organ damage, often with poor reversibility, has developed.^9^ Taken together, all the features mentioned above account for an extremely difficult early recognition or even clinical suspect of HES, I-HES in particular, and on time referral to a specialized centre. The referral itself is challenging due to the a not univocal specialist identification, due to the variability of physicians managing HES in different settings and including allergist/clinical immunologist, haematologist, internal medicine doctors, pulmonologist, rheumatologist. Under that perspective HES is not only rare but also orphan, this condition also referring to the poorly standardized approach in terms of personalized treatment identification and follow-up plan (timing, organ assessment), the last being challenging in the light of the multiple eosinophils-related pathobiological mechanisms and systemic/multi organ involvement in HES (Table 1).

**Table 1:** Challenges, unmet needs and potential strategies in HES practical management

	Common challenges		Potential recovery strategies
	in rare diseases	in “orphan” diseases	
Diagnosis	Heterogeneous clinical presentation	Difficult (early) diagnostic suspect	Increasing the overall clinicians' awareness
	Non-specific findings on physical exam, lab and imaging	Not univocal specialist identification	
	overlap with other entities	Referral	Implementing inter-hospitals network and second opinion
	Diagnosis/“phenotyping"	Implementing hospital-territory network	
Care	Multiorgan involvement	No standardized approach in terms of treatment and follow-up	optimising intra-hospital healthcare pathways
	Multiple pathobiological mechanisms		

Further translational and clinical research is needed to address the mentioned unmet needs, but on a practical ground increasing the overall clinicians’ awareness on HES and implementing healthcare pathways for HES patients represent a roadmap that every clinician might try to realize in his specific setting.

## Organizational perspectives – the Verona model

The Italian Government has recently established that rare diseases must represent a priority, in terms of research and clinical care (Law n. 175, 2021). Similarly, the United Nations have included the topic in the 76th General Assembly (December 16, 2021), highlighting the need for a strategic plan focused on the patients affected by rare diseases, in order to improve resources and opportunities for them.

**Fig. 4:**
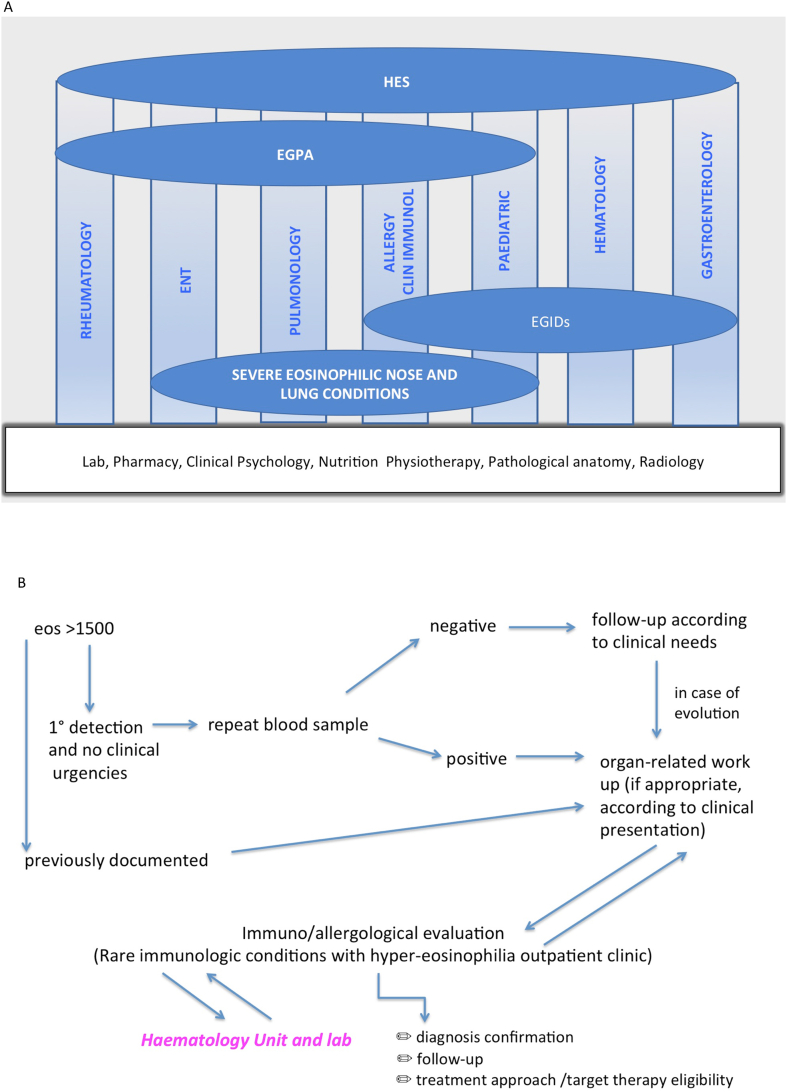
Panel A. Structure of the organizational model related to rare dysimmune conditions with hyper-eosinophilia at Verona Integrated University Hospital, including the core Units contributing to the multidisciplinary group (vertical bars), the common services shared by all the Units (horizontal bar) and the hypereosinophilic conditions managed by Units subgroups (horizontal ovals). EGIDS: eosinophilic disorders of the digestive tract. EGPA: eosinophilic granulomatosis with polyangiitis. HES: hypereosinophilic syndromes. Panel B. Standardized roadmap driving the approach to patients according to red-flags and criteria shared by the GEos (multidisciplinary Group on rare dysimmune conditions with hyper-Eosinophilia) Units at Verona Integrated University Hospital

As a local implementation, the Strategic and General Direction of Integrated University Hospital of Verona has promoted^53^ and finally approved by a formal deliberation^54^ the initiative to create a multidisciplinary Group on rare dysimmune conditions with hyper-Eosinophilia (GEos), which to the best of our knowledge, is the first institutionally formalized example in the field at least in Italy. The team-working model including different specialists is not new in the field of allergy and clinical immunology, which is characterised by a transversal approach by definition. As a novelty in respect of the traditional multidisciplinary approach, focused on one single disease or one organ still with systemic burden, in GEos we integrated all the specialists more likely to intercept patients with clinical manifestations attributable to hyper-eosinophilia, whether eosinophilic organ diseases or systemic eosinophil-driven dis-immune conditions. The specialists included in GEos (Fig. 4 Panel A) are not intended as a closed list, but a core group of clinicians actively contributing to HES recognition and management, which is in our opinion the first requirement to deal with I-HES. The first very practical aim of the MG institution was increasing the clinicians' knowledge and awareness about HES, as well as implementing intra-Unit and inter-Units referral by identifying a focused specialist in each Unit participating to the MG. The multidisciplinary discussion takes place in different formats, including patient's evaluation in the presence of different specialists together, or focused discussions of difficult cases among specialists only. However, a red flags system approach and a roadmap shared by all the GEos specialists (Fig. 4 Panel B) allows to tailor and optimize on a single-case basis the participation of the different clinicians to the multidisciplinary discussion, by avoiding to recruit all the Group for every case but at the same time to provide the patients with the needed expertise. On the other hand, the same-shared system harmonizes the specialists in the first approach and save time for the patient along the way of final diagnosis confirmation. More in detail, as summarized in Fig. 4 Panel B, a blood eosinophils count >1500 cells/microliter is considered the hallmark of a potential HES. In the case of a first detection, the same clinician prescribes a second sample according to the timing requested for hyper-eosinophilia diagnosis. If the condition is not confirmed, a follow-up according to clinical needs is planned.

If a second detection of blood hyper-eosinophilia occurs, or a previous one is already documented, the organ-related work up is performed, driven by the reason why the patient is visiting the clinician and looking for the evidence of eosinophilic organ condition. Once completed the single-specialist assessment, the patient is evaluated at the GEos outpatient clinic hosted in the Allergy Unit and Asthma Centre of our Hospital and the case further discussed with the referring physician, the haematologist and other specialists of the Group, or external ones if required. In addition, the molecular diagnosis is evaluated and planned with the Haematology Unit and Lab. Once the diagnosis is confirmed and haematological malignancies have been excluded, treatment approach is evaluated; off label or not-marketed options are explored if needed together with the Pharmacy Unit if needed. Follow-up visits are scheduled and each case regularly re-discussed following the evolution of the disease and the response to therapy.(Fig. 4 Panel B).

GEos Multidisciplinary group can also rely on the expertise of “transversal services”, including Lab, Pharmacy, Clinical Psychology, Nutrition, Physiotherapy, Pathological anatomy, Radiology, which serve as common basis for all the Units and Multidisciplinary Groups managing rare diseases within the Integrated University Hospital of Verona, according to an innovative organizational model focused on rare diseases (Fig. 4).^55^

## Conclusions

Hyper-eosinophilic syndromes, and even more I-HES, are at the centre of the current research to clarify underlying mechanisms and ameliorate the disease management. On practical grounds, the HES patient journey deserves a major focus in order to reduce the delay in final diagnosis, to optimize exams and procedures by avoiding unnecessary or un-appropriate investigations, to implement the referral to experienced Centres. It requires to develop organizational models aimed to integrate intra-hospital healthcare pathways, but also to consolidate the inter-hospitals networks sustaining counselling and second opinion services, and to connect hospitals with the first line territorial Health Care Professionals, including General Practitioners, Emergency rooms departments and Community Pharmacies. It should be considered as the first step to increase the general knowledge and expertise about rare immunological diseases with hyper-eosinophilia, and to shorten the overall journey from the identification of the affected patients to the evaluation of new target-therapies, without neglecting the complexity of the management of those conditions.

Implementing healthcare pathways for HES patients represent the doctors' journey that every clinician might try to realize in his specific setting, to finally improve the standard of care and patient's quality of life in HES.

## Abbreviations

ABPA: Allergic bronchopulmonary aspergillosis; CMR: cardiovascular magnetic resonance; CT: computes tomography; CRSwNP: chronic rhinosinusitis with nasal polyps; EGPA: Eosinophilic granulomatosis with polyangiitis; EGIDs: eosinophilic disorders of the digestive tract; EoE: eosinophilic esophagitis; EP: eosinophilic pneumonia; HES: Hypereosinophilic syndrome; I-HES: idiopathic subtype; L-HES: lymphocytic HES; M-HES: myeloid HES; TCR: T cell receptor.

## Funding

No funding for this work.

## Availability of data and materials

Not applicable.

## Authors’ contributions

MC, LFC, GS and MC conceived the article structure and drafted the manuscript. CA, FA, RB, FC, LDF, MDG, GF, SF, LF, PG, MK, GL, CM, GP, PP, MR, MS, CT, LT, ET contributed to the literary review and writing of specific manuscript paragraphs. All the authors read and approved the final version of the manuscript.

## Ethics approval

Not applicable.

## Authors’ consent for publication

All the authors provided consent to publication.

## Declaration of competing interest

The authors declare that they have no competing interests in relation to the topic of the manuscript.
